# First record of the antlion *Solterliber* Navás, 1912 in Italy (Neuroptera, Myrmeleontidae)

**DOI:** 10.3897/BDJ.12.e132510

**Published:** 2024-09-24

**Authors:** Davide Badano, Rebecca Funari, Filippo Di Giovanni

**Affiliations:** 1 Department of Life Sciences, University of Siena, Siena, Italy Department of Life Sciences, University of Siena Siena Italy; 2 NBFC, National Biodiversity Future Center, Palermo, Italy NBFC, National Biodiversity Future Center Palermo Italy

**Keywords:** island, distribution, lacewings, Mediterranean, Myrmeleontoidea, Sicily

## Abstract

**Background:**

Myrmeleontidae are conspicuous insects with moderate species diversity, which are reasonably well studied in Europe. However, dedicated samplings in the Mediterranean region carried out in the last years suggest that the state of knowledge of the representatives of this family occurring in outhern Europe is far from ideal. Over 40 species of antlions and owlflies are reported from Italy, of which a few are only known from the islands of the Sicilian Channel.

**New information:**

*Solterliber* Navás, 1912 is reported for the first time in Italy, based on specimens collected in Pantelleria Island in 2022 and 2023.

## Introduction

Myrmeleontidae, or antlions, are iconic insects mostly occurring in arid habitats. Their large size and unusual larval ethology have attracted the attention of naturalists since the dawn of scientific exploration (Aspöck and Aspöck 2007). Nevertheless, even in well-investigated Europe, for which several monographs dedicated to neuropterans exist, searches aimed at antlions in southern Europe have resulted in the discovery of new species or remarkable biogeographic findings (Pantaleoni and Badano 2012, Badano et al. 2016). Mediterranean islands represent particularly interesting areas, acting as stepping-stones between North Africa and Europe and often harbouring remarkable species (Massa 1995). Forty species of Myrmeleontidae (including ascalaphids) are present in Italy, of which four are only known from the small islands surrounding Sicily, i.e. *Bubopsisagrionoides* (Rambur, 1838), *Creoleonaegyptiacus* (Rambur, 1842), *Creoleongriseus* (Klug in Ehrenberg, 1834) and *Distoleonannulatus* (Klug *in* Ehrenberg, 1834) (Letardi 2005, Nicoli Aldini et al. 2012, Letardi and Badano 2017). Therefore, this region represents one of the most interesting areas for the biogeography of these insects at the national level.

The genus *Solter* Navás includes 36 species and is one of the antlion genera characteristic of the Western Palaearctic dry areas, with a few species present in the Afrotropical Region (Mansell 2013, Michel 2014, Oswald 2024). The type species, *Solterliber* Navás, 1912, is the only species of *Solter* known in Europe, although this genus reaches is highest species diversity in neighbouring areas, such as Central Asia, North Africa and the Middle East (Aspöck et al. 1980, Aspöck et al. 2001, Monserrat and Acevedo 2014). Here, we report the presence of *S.liber* in Pantelleria, an island located in the Sicilian Channel, which is also the first record of this genus and species for Italy.

## Materials and methods

Digital images of the specimen habitus were taken using a Canon EOS 600D camera equipped with Canon Photo lens MP-E 65 mm 1:2.8 and processed by Canon Digital Photo Professional. Morphological comparisons, measurements and images of anatomical details were carried out with a Zeiss Axio Zoom V16 microscope. The resulting images were processed with Zerene Stacker for photo stacking.

Specimen repository: DBC; Davide Badano research collection (Siena, Italy).

Classification system follows Machado et al. (2018).

The distribution map was made with the software R version 4.2.1 (R Core Team 2012), based on georeferenced data available from Michel (2014), Ábrahám (2017) and GBIF (2023). Identifications of GBIF records were checked before inclusion.

Genomic DNA was extracted from the leg of one of the specimens and preserved in ethanol. Mitochondrial DNA fragment Cytochrome Oxidase subunit I was amplified with the forward primer LCO1490 (5’- GGTCAACAAATCATAAAGATATTGG -3’) and the reverse primer HCO2198 (5’- TAAACTTCAGGGTGACCAAAAAATCA -3’) (Folmer et al. 1994). PCRs were executed in 25 μl reaction volume with 2.5 μl of DNA from each sample, 1.25 μl of both forward and reverse primers (10 μM), 2.5 μl of MgCl_2_ (25 mM), 2.5 μl of deoxynucleotides (dNTPs, 10 mM), 5 μl of Green GoTaq Flexi Buffer (Promega, US), 0.125 μl of GoTaq Flexi DNA polymerase (Promega, US) [5 u/μl] and 9.875 μl of sterile ddH_2_O. Amplifications were obtained in a T100 Thermal cycler (BioRad, US) Thermal Cycler, using the following conditions: (i) 95°C for 5 min; (ii) 35 cycles of 95°C for 1 min, 50°C for 1 min, 72°C for 90 s; (iii) 72°C for 7 min. The kit Wizard® SV Gel and PCR Clean-Up System (Promega, US) was employed for PCR products purification and their DNA concentration was assessed through a NanoDrop One (Thermo Scientific, US) device. The quality of PCR results and purifications was checked through 1% agarose gel. Samples were sequenced with the same primers and applying Sanger techniques at the core facility of BMR Genomics (Padua, IT) run by an automated DNA sequencer.

The obtained COI sequence was edited and checked with MEGA XI (Tamura et al. 2021). The barcode sequence was analysed through the integrated bioinformatics platform Barcode of Life Data (BOLD) System (Ratnasingham and Hebert 2007) to test the morphology-based species identification.

COI sequences obtained from GenBank (Table 1) were aligned using the ClustalW programme (Thompson et al. 1994) implemented in MEGA XI. The Neighbour-joining (NJ) cluster analysis was run with MEGA XI, selecting the default parameters.

## Taxon treatments

### Solter liber

Navás, 1912

D6177B02-8CD4-5707-AF04-0BB5BD1480E0

PQ045664

#### Materials

- **Type status:** Other material. **Occurrence:** recordedBy: Filippo Di Giovanni; individualCount: 1; sex: male; lifeStage: adult; occurrenceID: 7F2F4D28-D50D-5F07-9D0E-E77D35AD77EF; **Taxon:** scientificName: Solterliber; kingdom: Animalia; phylum: Arthropoda; class: Insecta; order: Neuroptera; family: Myrmeleontidae; genus: Solter; specificEpithet: liber; scientificNameAuthorship: Navás, 1912; **Location:** country: Italy; countryCode: IT; stateProvince: Sicily; county: Trapani; municipality: Pantelleria; locality: Contrada Khaddiuggia; verbatimElevation: 115 m; verbatimCoordinates: 36.8228389 11.9759722; decimalLatitude: 36.8228389; decimalLongitude: 11.9759722; geodeticDatum: WGS84; **Identification:** identifiedBy: Davide Badano; dateIdentified: 2022; **Event:** samplingProtocol: light; eventDate: 13-08-2022; **Record Level:** collectionID: DBC
- **Type status:** Other material. **Occurrence:** recordedBy: Filippo Di Giovanni; individualCount: 1; sex: female; lifeStage: adult; occurrenceID: BF3C89B3-F6C5-5E0F-8B10-B4D4F8DE155D; **Taxon:** scientificName: Solterliber; kingdom: Animalia; phylum: Arthropoda; class: Insecta; order: Neuroptera; family: Myrmeleontidae; genus: Solter; specificEpithet: liber; scientificNameAuthorship: Navás, 1912; **Location:** country: Italy; countryCode: IT; stateProvince: Sicily; county: Trapani; municipality: Pantelleria; locality: Contrada Khaddiuggia; verbatimElevation: 115 m; decimalLatitude: 36.8228389; decimalLongitude: 11.9759722; geodeticDatum: WGS84; **Identification:** identifiedBy: Davide Badano; dateIdentified: 2023; **Event:** samplingProtocol: light; eventDate: 22-08-2023; **Record Level:** collectionID: DBC

#### Distribution

**Figure 1.**
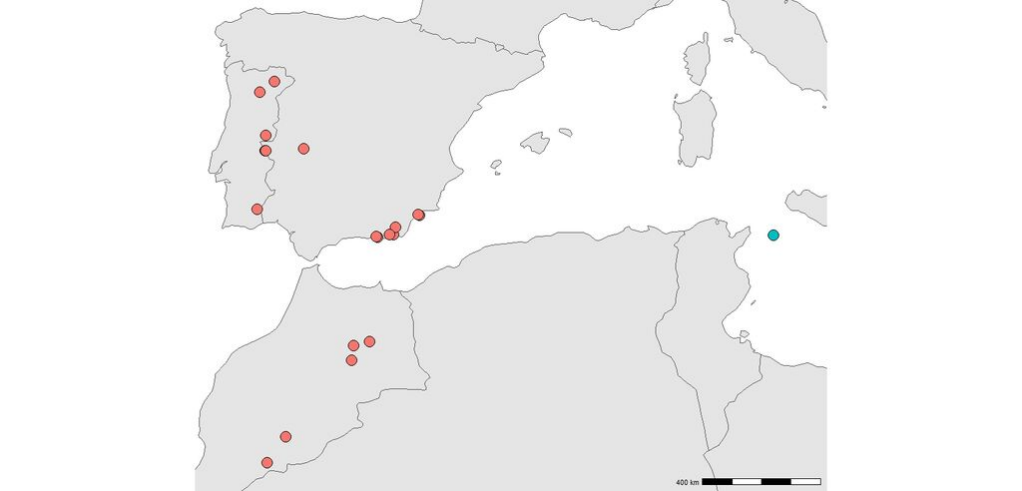
Distribution of *Solterliber* Navás, 1912, based on available georeferenced records, highlighting the new record from Pantelleria.

*Solterliber* is definitely known from the Iberian Peninsula (Portugal, Spain) and northern Africa (Morocco) (Fig. 1) (Michel 2014, Monserrat and Acevedo 2014, Ábrahám 2017). Older records from Algeria (Navás 1918, Navás 1919, Navás 1928), Egypt (Navás 1926), Libyia (Navás 1929, Esben-Petersen 1936) and Tunisia (Navás 1930), predating the genus revision by Michel (2014), as well as those from the Middle East (Israel, Turkey) (Simon 1979, Ari and Kiyak 2000), need to be revised. This species is widespread in the arid habitats of the Iberian Peninsula, although it remains rare (Monserrat and Acevedo 2014).

#### Biology

**Figure 2.**
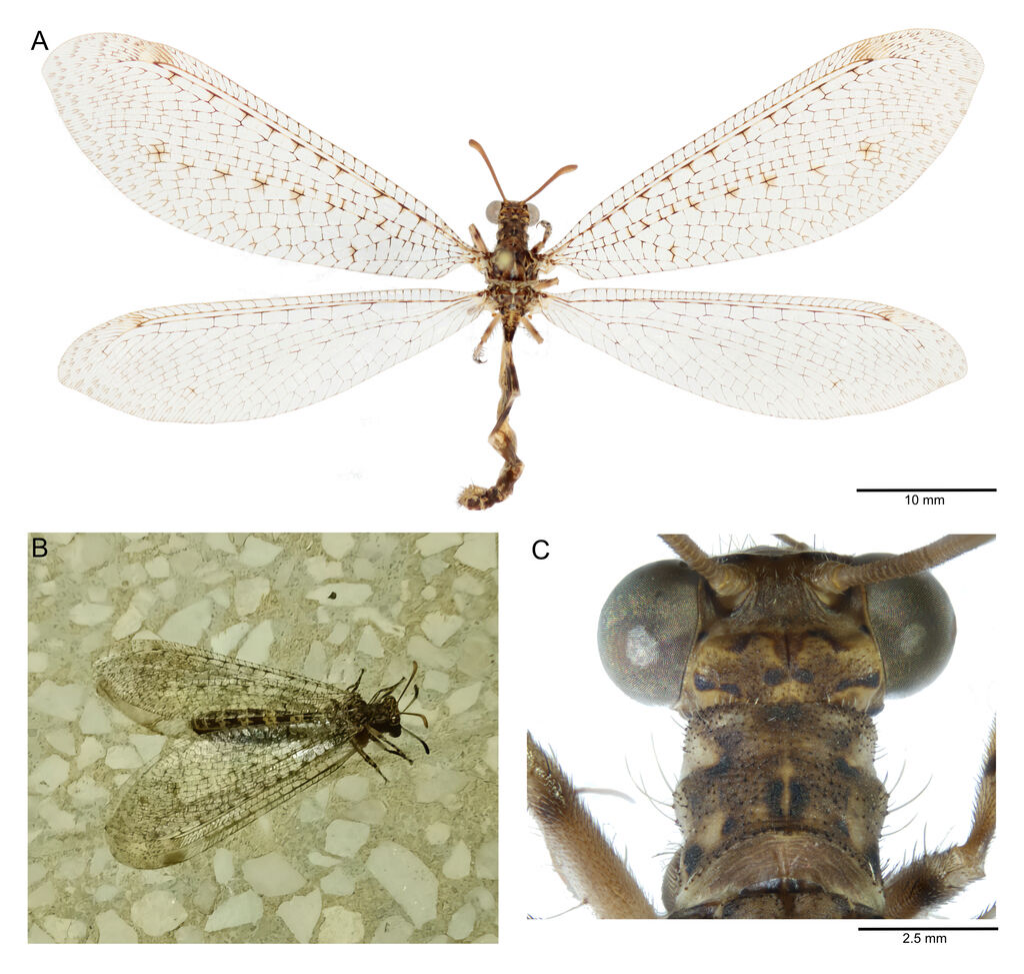
*Solterliber* Navás, 1912, male specimen collected in Pantelleria, Contrada Khaddiuggia, in 2022: **A** habitus; **B** live specimen at light; **C** detail of head and pronotum.

*Solterliber* inhabits dry Mediterranean habitats and desert-like environments. This antlion is usually found in rocky habitats with scattered vegetation covering (Monserrat and Acevedo 2014). The adults are characterised by a brown, strongly patterned colouration characteristic of antlions commonly resting on rock surfaces (Fig. 2). The larvae, like other congeners, are found under overhangs in rocky outcrops where they remain motionless, buried under a thin layer of sand, acting as ambush hunters (Badano et al. 2014).

#### Notes

The present findings are the first records of genus *Solter* in Italy, representing a significant addition to the lacewing fauna of Pantelleria.

## Analysis

### DNA barcoding

**Figure 3.**
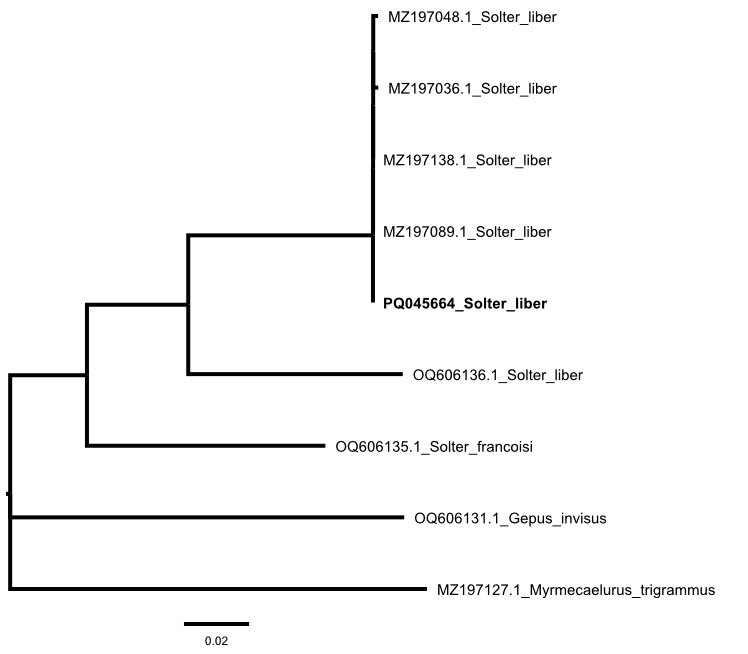
Phylogenetic relationships of cytochrome c oxidase subunit I COI in the genus *Solter* reconstructed with the Neighbor-Joining method. The sequence of the specimen of *Solterliber* from Pantelleria (PQ045664), in bold, clusters with the available sequences obtained from Portuguese specimens.

The DNA barcoding resulted in a sequence of 695 bp, which was deposited on GenBank and accessible under the accession number PQ045664. The comparison of our sequence with those present in GenBank through BLAST revealed a 100.0% similarity with *Solterliber* (Fig. 3), confirming the morphology-based identification. In the NJ analysis, our sequence clustered together with the available sequences of *S.liber* specimens from Portugal.

## Discussion

The volcanic island of Pantelleria is closer to North Africa than to Sicily, being 65 km from the Tunisian coast (Ras el-Mustafà) and 95 km from the Sicilian coast (Capo Granitola). However, from a biogeographic point of view, the insect fauna of this island shows stronger affinities with that of the Italian peninsula than to North Africa, likely due to the complex paleogeography of the Sicilian Channel (Massa 1995). Indeed, during the Last Glacial Maximum, the currently submerged Adventure Plateau formed a peninsula connected to Sicily, which allowed terrestrial species to colonise Pantelleria after volcanic eruptions (Lodolo and Ben-Avraham 2015, Massa et al. 2022). Few species of Myrmeleontidae are known from Pantelleria, i.e. *Macronemurusappendiculatus* (Latreille, 1807), *Distoleonannulatus* and *Creoleonlugdunensis* (Villers, 1789) (Nicoli Aldini et al. 2012). Moreover, on the island, two Sicilian endemic species are present: the antlion *Myrmeleonpunicanus* Pantaleoni and Badano, 2012 and the owlfly *Libelloidessiculus* (Angelini, 1827) (DBC), again supporting the close biogeographic association between Pantelleria and Sicily (Pantaleoni and Badano 2012). On the other hand, the finding of *Solterliber* in Pantelleria is emblematic because this species is not present in the Italian Peninsula or Sicily, suggesting that it colonised Pantelleria from North Africa instead. *Solterliber* is strictly associated with dry habitats; therefore, its ability to cross over 60 km of open sea, even if likely carried out by winds, is remarkable and hints at unrecognised dispersal abilities. *Solterliber* represents an interesting addition to a small number of myrmeleontid species that are only present in the islands of the Sicilian Channel for the Italian territory.

## Supplementary Material

XML Treatment for Solter liber

## Figures and Tables

**Table 1.** COI sequences of Myrmeleontidae obtained from GenBank (NCBI) used to run the analysis.

**GenBank accession number**	**Species**	**Geographic provenance**	**Reference**
MZ197127	*Myrmecaelurustrigrammus* (Pallas, 1771)	Portugal	(Oliveira et al. 2021)
OQ606131	*Gepusinvisus* Navás, 1914	Morocco	(Hévin et al. 2023)
OQ606135	*Solterfrancoisi* Michel, 2014	Morocco	(Hévin et al. 2023)
OQ606136	*Solterliber* Navás, 1912	Morocco	(Hévin et al. 2023)
MZ197036	*Solterliber* Navás, 1912	Portugal	(Oliveira et al. 2021)
MZ197089	*Solterliber* Navás, 1912	Portugal	(Oliveira et al. 2021)
MZ197138	*Solterliber* Navás, 1912	Portugal	(Oliveira et al. 2021)
MZ197048	*Solterliber* Navás, 1912	Portugal	(Oliveira et al. 2021)
PQ045664	*Solterliber* Navás, 1912	Italy, Pantelleria	This study
